# Laser Applications in Wound and Scar Management Post-Mohs Micrographic Surgery: A Systematic Review

**DOI:** 10.1177/12034754241227629

**Published:** 2024-02-14

**Authors:** Michelle Le, Chaocheng Liu, Owen D. Luo, Delaram Shojaei, Christopher D. Sibley

**Affiliations:** 1 Division of Dermatology, McGill University Health Centre, Montreal, QC, Canada; 2 Department of Dermatology and Skin Science, University of British Columbia, Vancouver, BC, Canada; 3 Department of Medicine, McGill University, Montreal, QC, Canada; 4 Department of Medicine, University of British Columbia, Vancouver, BC, Canada; 5 Laserderm, Ottawa, ON, Canada

**Keywords:** laser, surgery, wound

## Abstract

Mohs micrographic surgery (MMS) can lead to complications such as scarring and delayed wound healing, particularly in sensitive areas such as the face, neck, and chest. This study aims to assess the evidence regarding the use of lasers post-MMS for wound healing and scar revision. A comprehensive systematic review of the literature was performed using databases including MEDLINE, PubMed, EMBASE, Web of Science, Cochrane Library, and CINAHL from inception until July 25, 2022. A total of 2147 unique studies were identified, from which 17 were included in the analysis. A total of 17 studies reported applications of lasers with favourable efficacy including wound healing (n = 1), resurfacing of full-thickness skin grafts and split-thickness skin grafts (n = 4), periscar telangiectasias (n = 1), functional scar contractures (n = 2), and scar texture (n = 9). Minimal adverse effects were reported with the use of lasers post-MMS. Overall, the use of lasers post-MMS is a safe and well-tolerated option for scar revision with high patient satisfaction and is less invasive than surgical interventions.

## Introduction

Mohs micrographic surgery (MMS) is a specialized technique used for high-risk skin cancers, involving precise layer-by-layer removal and histopathological examination.^1,2^ However, MMS often leads to scarring as a common adverse outcome of the procedure.^3^ Scarring, especially on sensitive areas such as the face, neck, chest, and genital areas, has been associated with higher incidences of post-traumatic stress disorder and depression, marital problems, unemployment, and alcoholism.^4,5^ Further, most patients underestimate the length and extent of post-surgical scars.^6^ Thus, examination of scars post-MMS is an important topic.

Scars may be hypertrophic, atrophic, or sclerotic. For post-MMS complications, persistent scar erythema, keloid formation, and hypertrophy have been identified as the most common.^7-9^ In addition to dyspigmentation and improperly healed full-thickness skin grafts (FTSGs) and split-thickness skin grafts (STSGs), scars can contribute to a loss of functionality, pain and discomfort, poor cosmesis, delayed wound healing, infections, graft failures, deformities, and contractures.^10-12^ Contractures may also result in ectropion or eclabium and other functional issues in gross motor skills, delayed walking, and painful plantar skin.^13^ Therefore, proper treatment of post-MMS scarring is essential to the appropriate care and management of patients.

Treatment of scars may include silicone gel dressings, dermabrasion, chemical peels, and surgical revision.^7,14^ Lasers have also been suggested as a safe, effective, and minimally invasive procedure for scar revision. Recent advances in fractional energy-based technology have promoted an increase in the applications of lasers for post-MMS scar revision and wound healing.^15,16^ However, there is a lack of systemic evaluation of the level of evidence for these interventions.^7^ Thus, the objective of this systematic review is to evaluate the efficacy and safety of laser devices to promote wound healing and treat post-MMS scars.

## Methods

Following the PRISMA (Preferred Reporting Items for Systematic Reviews and Meta-Analyses) guideline, a comprehensive systematic review was conducted on articles from databases including MEDLINE, PubMed, EMBASE, Web of Science, Cochrane Library, and CINAHL, from inception up to July 25, 2022. The search terms “laser,” “Mohs,” “MMS,” “Mohs micrographic surgery,” and “chemosurgery” were utilized to identify studies investigating laser applications in post-MMS management. Please see Supplementary Material for the full search strategy. In addition to database searches, references of pertinent articles were screened for additional studies. Three reviewers independently screened titles, abstracts, and full texts using predefined inclusion and exclusion criteria. The review encompassed original clinical studies, conference abstracts, case reports, case series, observational studies, and randomized controlled trials (RCTs) in English or French while excluding review articles. Primary outcomes covered the laser device specifics, treatment parameters, treatment purpose, and clinical outcomes, with adverse effects as secondary outcomes. Data extraction utilized a pre-developed standardized form based on the outcomes of interests, with studies categorized based on the laser’s application. The extracted data were verified by a second reviewer. Higher-evidence RCTs and comparative studies were analyzed separately. The quality assessment involved using the Joanna Briggs Institute Critical Appraisal Checklist for Case Reports, Case Series (Supplementary Table S1), and Cochrane Risk of Bias Tool for RCTs (Supplementary Table S2).

## Results

### Search Results

A total of 2147 unique studies were identified of which 17 were included in the analysis (Supplementary Figure S1). A summary of study characteristics, treatment modalities, and outcomes can be found in Supplementary Table S3.

### Wound Healing

One case report by Fancher et al described the use of lasers as an aid to wound healing in a Marjolin ulcer complicated by *mycobacterium fortuitum* infection. They described a 5.0 cm × 5.8 cm post-MMS defect of the lower leg with extension to the fascia which healed via secondary intention. A pulsed dye laser (PDL; 595 nm) was used to prevent hyper-granulation, and expedite wound healing after treatment with antibiotics and processed dehydrated human amnion-chorion membrane allograft. Substantial granulation tissue covering the entire fascia was observed on day 22 and by day 36, the defect had filled in with granulation tissue. By day 56, the wound fully re-epithelialized with a residual scar measuring 2.0 cm × 2.0 cm.^17^

### FTSG Resurfacing

Four case reports and case series with a total of 99 patients described resurfacing after FTSG; the majority of the procedures occurred on the nose.^18-21^ Two studies used an ablative fractional laser (AFL) with carbon dioxide (CO_2_; 10,600 nm) 2 months post-MMS, which improved the transition of the FTSG to the surrounding skin with respect to scar texture, contour, and colour-match.^18,19^ One study also observed improved FTSG cosmesis after using the non-ablative fractional laser (NAFL; 1540 nm) 4 years post-MMS.^20^ Another study of 74 patients used the AFL with CO_2_ immediately after MMS and reported “acceptable” to “excellent” results in all patients by physicians and improved patient satisfaction regarding scar cosmesis.^21^

### Periscar Telangiectasia

One article reviewed laser applications for periscar telangiectasia. Villeneuve-Tang et al evaluated the use of one treatment of potassium titanyl phosphate laser (KTP; 532 nm) for periscar telangiectasia 3 months post-MMS on the nose and forehead. Of the 6 patients who had a single treatment session, 2 had minimal clearance (0%–25% clearance), 3 had good clearance (50%–75% clearance), and 1 had excellent clearance (75%–100% clearance).^22^

### Functional Scar Contractures

Two case series with a total of 14 patients by Ezra et al, in 2016 and 2014, described the use of microsecond neodymium-doped yttrium aluminum garnet laser (Nd:YAG; 1064 nm) for management of ectropion and eclabium, secondary to scar contractures post-MMS.^23,24^ Of 14 patients who underwent microsecond Nd:YAG laser treatment, 1 patient showed improvement of ectropion and 7 experienced complete resolution of ectropion or eclabium, demonstrating a 50% overall success rate.^23,24^ Regarding the frequency of treatments, 1 to 3 treatments at 1- to 2-month intervals were reported to have good clinical outcomes for patients. While transient erythema was noted 1 to 2 hours post-treatment, no recurrence nor adverse events were noted.^23,24^

### Textural Scar Changes

Nine articles reviewed laser applications for textural scar changes. Studies by Chen et al, Ezra et al, Kunishige et al, and Cohen report treating hypertrophic scars with PDL, microsecond Nd:YAG laser, NAFL, and AFL with Er:YAG (2940 nm), respectively.^23,25-27^ In Chen et al’s study, a hypertrophic scar on the leg was treated with 2 monthly sessions of PDL at 3 months post-MMS.^26^ The authors reported a 5-point improvement on the Vancouver Scar Scale (VSS), which is a scale that grades scar characteristics including vascularity, pigmentation, pliability, and scar height.^28^ A 20-point improvement was also observed on the patient Scar Assessment Scale (SAS), and a 21-point improvement on the observer SAS.^29^ While the patient SAS scores scar characteristics including pain, itch, thickness, colour, stiffness, and irregularity; the observer SAS scores scar vascularity, pigmentation, texture, thickness, pliability, and surface area. Two years after the surgery, the authors reported that the scar had almost disappeared.^28^ In the study by Ezra et al, 1 to 3 sessions of microsecond Nd:YAG treatment, 1 to 2 months apart, were used to treat hypertrophic scars in 5 patients.^23^ Among these patients, all had improved appearance of their scars and all patients were pleased with their results. Side effects were limited to transient erythema post-laser treatment which lasted 1 to 2 hours.^23^ In the case series of 6 patients by Kunishige et al, hypertrophic scars were treated with 6 to 10 passes of NAFL over the scar with an average of 3 sessions each.^25^ Nearly all patients achieved at least a 50% overall improvement in scar erythema, pigment, and height using a quartile grading scale. Moderate to severe erythema and edema were observed and typically resolved within 24 to 48 hours.^25^ In Cohen’s case report, a scar on the right cheek displaying telangiectasias, hypo- and hyper-pigmentation, and isolated fibrotic lines, 2 to 3 months post-MMS, was treated with 3 sessions of 2 passes of AFL with Er:YAG.^27^ The laser treatments were well-tolerated and elicited significant improvement in scar pigmentation, vascularity, and texture.^27^

#### Microsecond Nd:YAG laser and NAFL

The use of lasers to improve atrophic scars was evaluated by Ezra et al, using the microsecond Nd:YAG laser, and Schulz and Walling and Kunishige et al, using the NAFL.^23,25,30^ In the study by Ezra et al, 3 patients with atrophic and sclerotic scars demonstrated improved cosmesis and patient satisfaction with 1 to 3 sessions of microsecond Nd:YAG laser treatment, 1 to 2 months apart.^23^ Schulz and Walling’s case report described the successful use of NAFL for an atrophic scar after secondary intention healing of the right nasal ala, 8 months post-MMS.^30^ The scar was treated with 5 monthly sessions, was reported to be nearly imperceptible at 1 month post-treatment, and remained stable 18 months post-treatment.^30^ Kunishige et al, described a case series of 6 patients which included treatment of atrophic scars with an average of 3 sessions of 6 to 10 passes of NAFL.^25^ Nearly all patients achieved at least a 50% overall improvement in scar erythema, pigment and height using a quartile grading scale. Similar to the case report by Schulz and Walling, moderate to severe erythema and edema were observed and typically resolved within 24 to 48 hours.^25^

#### PDL and AFL with CO_2_

In 2016, Cohen and Geronimus published a randomized control trial evaluating PDL (treatment arm 1), AFL with CO2 (treatment arm 2), combined PDL and AFL with CO_2_ (treatment arm 3), and a split-scar study (treatment arm 4) where one-half of the scar was untreated (control) while the other half was treated with AFL with CO_2_ immediately after surgery and then 3 combined PDL and AFL with CO_2_ treatments. These treatments were done for 25 patients with scars on various parts of the body (n = 22 for face, n = 2 for chest, n = 1 for arm).^31^ Three (treatment arms 1, 2, and 3) or 4 (treatment arm 4) treatments were conducted 6 to 8 weeks apart and patients were evaluated using the VSS and Global Evaluation Response Scale. Improvement in scar cosmesis was observed in all treatment arms. In treatment arm 1, 2 patients had complete scar clearance and 4 patients had scars, which were almost cleared. In treatment arm 2, 4 patients’ scars almost cleared and 2 had moderate improvement. In treatment arm 4, 2 scars were almost cleared, 3 had marked improvement, and 1 had moderate improvement, compared to the control. Authors observed more improvement in vascularity following PDL than AFL with CO_2_, while AFL with CO_2_ demonstrated greater improvement in pigmentation. Among patients in all treatment groups, 74% to 91% reported none to moderate pain from the laser treatment. No adverse events were reported.^31^

Similarly, in a study by Kim et al, a prospective randomized, evaluator-blinded, comparative split-scar study with 14 patients was conducted where AFL with CO_2_ was used for one-half of participants’ scars and PDL for the other half of participants’ scars, 2 weeks post-MMS.^32^ The scars were on the patients’ faces (n = 12) and abdomens (n = 2). Both PDL and AFL with CO_2_ yielded statistically significant improvements based on overall VSS (*P* < .05), where an overall 3.21-point improvement was observed with PDL, and a 2.50-point improvement was observed with AFL with CO_2_. However, when comparing the 2 laser treatments, there was no statistically significant difference in improvement outcomes based on overall VSS scores. AFL with CO_2_ was more effective than PDL in the improvement of pliability and thickness. PDL was superior to AFL with CO_2_ in the improvement of vascularity and pigmentation. All participants reported post-therapy erythema and mild edema that resolved within 1 week. Post-treatment hyperpigmentation developed in 2 patients who received AFL with CO_2_.^32^

Sobanko et al conducted another prospective randomized, evaluator-blinded, comparative split-scar study, focusing on the use of AFL with CO_2_ for scar treatment of 20 patients. In this study, one treatment of AFL with CO_2_ was administered to one-half of the participants’ linear scars, and the treatment was performed 6 to 7 days post-MMS.^33^ The clinical outcomes were assessed using VSS and the patient cosmetic visual analogue scale (CVAS). No statistically significant difference was observed in VSS (*P* = .31). However, a statistically significant difference was observed in patient CVAS (*P* = .002), 12 weeks post-MMS. All patients tolerated the laser treatment well with no adverse events reported.^33^

#### Full-field erbium laser

A different study by Annolik et al conducted a comparative retrospective chart review study that compared the cosmetic outcomes of 10 patients who received full-field erbium laser treatments to 10 patients who did not receive laser treatment. The treatment was applied to wound edges immediately post-MMS of facial non-melanoma skin cancers.^34^ Patients undergoing laser ablation displayed less noticeable scars with less evident vertical “drop-off” and shadowing at the border of uninvolved to involved skin 3 to 4 months postoperatively as scored by blinded, non-treating dermatologists and the Primos optical tomography imaging system.^34^

Lastly, Tierney et al conducted a randomized, evaluator-blinded, comparative split-scar study where NAFL was used for one-half of participants’ scars and PDL for the other one-half of participants’ scars, a minimum of 2 months post-MMS.^35^ A total of 15 scars were included where 7 scars were on the face, 5 were on the chest, 2 were on the neck, and 1 was on the back. Scar dyspigmentation, thickness, texture, and overall cosmetic appearance were assessed on a 5-point grading scale. Overall mean improvement was 75.6% (range 60%-100%) in the NAFL group which is significantly better in comparison to 53.9% in the PDL group (range 20%-80%). Side effects with PDL were mild and limited to transient erythema and purpura.^35^

## Discussion

Careful decision-making is essential in choosing the proper laser device for the treatment of post-MMS scars. To the best of our knowledge, this is the first systematic review summarizing the utility of lasers for a variety of post-MMS complications. These advantages extend to improved wound healing, resurfacing of FTSGs and STSGs, and revising periscar telangiectasias, functional scar contractures, and scar texture. The PDL (595 nm), AFLs (CO_2_; 10,600 nm or Er:YAG; 2940 nm), and NAFL (1550 nm) were the most commonly used for scar revision, at various times post-MMS ranging from immediately post-procedure to up to 4 years later. The PDL was successfully used to improve scar hypertrophy, vascularity, and pigmentation as well as expedite wound healing via secondary intention. The AFLs were successful in improving the transition from the FTSG to the surrounding skin, and in improving scar pigmentation, pliability, and thickness. The NAFL was shown to have utility in resurfacing skin grafts including FTSG and STSG, revising atrophic scars as well as improving scar hypertrophy, erythema, pigmentation, thickness, and texture. The microsecond Nd:YAG laser treatment led to improved cosmesis and patient satisfaction in atrophic and sclerotic scars, while NAFL demonstrated success in achieving near imperceptibility and stable results in atrophic scars. Both modalities showed promising implications for enhancing cosmetic outcomes and patient satisfaction in scar management. Although studies were limited, evidence suggested that PDL is preferred for hyperpigmentation, erythema, and periscar telangiectasias, and NAFL and AFL for irregular textures from hypertrophic or atrophic scars.

The wavelength of PDL permits it to target oxyhemoglobin as its chromophore to induce localized thermal injury of red blood cells in the scar to selectively mitigate postoperative erythema and telangiectasias.^36^ On the other hand, AFL lasers target water as a chromophore, which exists in the extracellular tissue, and results in nonselective tissue injury with the goal of subsequent re-epithelization and remodelling.^37^ While there are studies comparing NAFL or AFL to PDL post-MMS, there is a need for head-to-head studies that compare PDL (595 nm) to its counterpart, KTP (532 nm), and NAFL (1550 nm) to its counterpart, AFL (CO_2_; 10,600 nm or Er:YAG; 2940 nm). This comparison is needed to determine the preferred laser modality to address scars with high vascularity or textural changes post-MMS, respectively.^37^

Lasers post-MMS are generally well-tolerated, with studies documenting common adverse events that span the spectrum of laser delivery modalities including mild-moderate procedural pain and postprocedural erythema and edema that last up to 1 week after treatment.^38,39^ Rare adverse events that are of note include crusts, purpura, and vesicles that last up to 4 days post-treatment of NAFL and post-treatment hyper- and hypopigmentation for patients who received AFL with CO_2_ laser treatments. These described adverse pigmentary alterations with AFL are related to the increased risk for nonselective thermal injury of AFLs.^40^ The diffuse thermal injury effects of AFLs can be mitigated by NAFLs, which generate microscopic columns of thermal injury without tissue destruction.^41^ It is paramount to use the appropriate wavelengths of laser energy for the specific scar target, which must be delivered with enough fluence to affect the target without collateral damage/adverse effects.^41^

There are several limitations among the studies included in this systematic review. Most of the studies were observational studies with small sample sizes, potentially due to the financial constraints associated with receiving laser therapy and limited access to specialized equipment and trained healthcare professionals.^42,43^ Additionally, there are significant heterogeneities among the studies, stemming from variations in the area on which treatment was applied, treatment timing and number of sessions, laser device parameters, and outcome assessment tools; thus, it is difficult to pool the data and subjective assessment of data may have resulted in outcome bias. Moreover, the availability of diverse laser devices varies across different countries, which further contributes to the heterogeneity in the analyzed literature.^44,45^ Lastly, laser-related complications heavily depend on the Fitzpatrick skin phototypes, a classification system based on patients’ melanin content and response to sun exposure, and patients with lighter skin types may be more susceptible to adverse reactions following post-MMS laser therapy; however, such data was rarely reported.^46^

## Conclusion

This systematic review provides a comprehensive summary of a variety of applications of laser for patients post-MMS to address both cosmetic and functional concerns. The review emphasizes the challenges posed by complications, particularly scar-related issues, which are a common occurrence post-MMS and can result in substantial patient distress, impaired function, and associated morbidities. Overall, laser therapy post-MMS is a safe and well-tolerated option with high patient satisfaction, and it is relatively less invasive than surgical scar revision. However, there is a lack of a control group for most studies, and the sample size of the randomized control studies is relatively small. Further studies with more consistent outcome assessment tools will be valuable in helping clinicians develop specific protocols and treatment regimens to offer more effective minimally invasive interventions to manage common complications resulting from MMS.

## Supplemental Material

sj-docx-1-cms-10.1177_12034754241227629 – Supplemental material for Laser Applications in Wound and Scar Management Post-Mohs Micrographic Surgery: A Systematic Review Supplemental material, sj-docx-1-cms-10.1177_12034754241227629 for Laser Applications in Wound and Scar Management Post-Mohs Micrographic Surgery: A Systematic Review by Michelle Le, Chaocheng Liu, Owen D. Luo, Delaram Shojaei and Christopher D. Sibley in Journal of Cutaneous Medicine and Surgery
