# Supplementary material for: Laser Applications in Wound and Scar Management Post-Mohs Micrographic Surgery: A Systematic Review
Source: J Cutan Med Surg. 2024 Feb 14;28(2):167–72. doi: 10.1177/12034754241227629 (PMC11015716; doi:10.1177/12034754241227629)
Supplement: sj-docx-1-cms-10.1177_12034754241227629 – Supplemental material for Laser Applications in Wound and Scar Management Post-Mohs Micrographic Surgery: A Systematic Review [file sj-docx-1-cms-10.1177_12034754241227629.docx]

**Supplementary Material**

**Search Strategy**

MEDLINE via PubMed: (chemosurgery OR "Mohs Surgery"[Mesh] OR "mohs micrographic surgery" OR mohs* OR "MMS") AND ("Lasers"[Mesh] OR laser* OR "Phototherapy"[Mesh]) -> 988 results

EMBASE via Ovid: 1. exp mohs micrographic surgery/ 2. MMS.mp 3. Moh*.mp 4. exp chemosurgery/ 5. 1 or 2 or 3 or 4 6. laser*.mp 7. exp laser/ 8. exp phototherapy/ 9. 6 or 7 or 8 10. 6 and 9 -> 946 results

CINAHL: ((MH "Microsurgery+") OR “MMS” OR “chemosurgery” OR “mohs micrographic surgery” OR mohs) AND ((MH "Laser Therapy+") AND ((MH "Phototherapy+") OR (MH "Lasers+") OR laser*) -> 1125 results

Web of Science: 1. (((TS=(chemosurgery)) OR TS=(MMS)) OR TS=(mohs micrographic surgery)) OR TS=(moh*) 2. (TS=(laser*)) OR TS=(phototherapy) 3. 1 AND 2 -> 729 results

Cochrane Library: 1. MeSH descriptor: [Mohs Surgery] explode all trees 2. chemosurgery 3. MMS 4. moh*

5. mohs micrographic surgery 6. #1 or #2 or #3 or #4 or #5 7. MeSH descriptor: [Lasers] explode all trees 8. MeSH descriptor: [Phototherapy] explode all trees 9. laser* 10. #7 or #8 or #9 11. #6 and #10 -> 231 trials, 11 protocols, 125 cochrane reviews

**Table S1. Quality Appraisal via JBI Critical Appraisal Checklist for Case Reports and Case Series**

| **Study** | **Study Design** | **Patient demographic characteristics were described** | **Patient history and clinical condition were clearly described** | **Objective assessment methods and results were clearly described** | **Intervention was clearly described** | **Post intervention clinical condition was clearly described** | **Adverse events were clearly described** | **Take-away lessons were provided** | **Overall appraisal** |
| --- | --- | --- | --- | --- | --- | --- | --- | --- | --- |
| Fancher 2015^17^ | Case report | Yes | Yes | No | Yes | Yes | No | Yes | Include |
| Worley 2019^18^ | Case report | Yes | Yes | No | Yes | Yes | No | Yes | Include |
| Forbat 2017^19^ | Case series | Yes | Yes | Yes | Yes | Yes | No | Yes | Include |
| Ammirati 2001^21^ | Case series | No | Yes | Yes | Yes | Yes | Yes | Yes | Include |
| Villeneuve-Tang 2018^22^ | Case series | Yes | Yes | Yes | Yes | Yes | Yes | Yes | Include |
| Ezra 2016^23^ | Case series | Yes | Yes | Yes | Yes | Yes | Yes | Yes | Include |
| Ezra 2014^24^ | Case series | Yes | Yes | Yes | Yes | Yes | Yes | Yes | Include |
| Chen 2020^26^ | Case report | Yes | Yes | Yes | Yes | Yes | No | Yes | Include |
| Kunishige 2010^25^ | Case series | No | Unclear | Yes | Yes | Yes | Yes | Yes | Include |
| Cohen 2013^27^ | Case report | Yes | Yes | No | Yes | Yes | No | Yes | Include |
| Schulz 2010^30^ | Case report | Yes | Yes | No | Yes | Yes | Yes | Yes | Include |
| Anolik 2011^34^ | Retrospective chart review | No | Yes | Yes | Yes | Yes | Yes | Yes | Include |

**Table S2. Quality Appraisal of Randomized Control Trials via Cochrane Risk of Bias Tool**

| **Study** | **Bias from randomization process** | **Bias due to deviations from intended intervention** | **Bias due to missing outcome data** | **Bias in the measurement of the outcome** | **Bias in the selection of reported results** | **Overall bias** |
| --- | --- | --- | --- | --- | --- | --- |
| Verhaeghe 2013^20^ | Low | Low | Low | Low | Low | Low |
| Cohen 2016^31^ | Low | Low | Low | Low | Low | Low |
| Kim 2014^32^ | Low | Low | Low | Low | Low | Low |
| Sobanko 2015^33^ | Low | Low | Low | Low | Low | Low |
| Tierney 2009^35^ | Low | Low | Low | Low | Low | Low |

**Table S3. Summary of studies using lasers for wound healing and scar revision post-Mohs surgery**

| **Author Year & Study Design (sample size N)** | **Scar Characteristics** | **Treatment** | **Device and Treatment parameters** | **Outcome Assessment Tool and/or Treatment Endpoint** | **Outcome** | **Adverse Effects** |
| --- | --- | --- | --- | --- | --- | --- |
| **Wound Healing** | | | | | | |
| Fancher 2015^17^  Case report (N=1) | 5.0 x 5.8 cm defect of the lower leg with extension to fascia | PDL (595 nm) with wound allograft of processed dehydrated human amnion-chorion membrane 1-week post-MMS | EpiFix; MiMedx  *Energy:* 8J  *Spot size:* 5-mm  *Pulse duration:* 6ms  *No. of treatments:* 4 at 2-week intervals | N/A | Substantial granulation tissue covering the entire fascia at Day 22. At Day 36, the defect had completely filled in with granulation tissue. At Day 56, the wound fully re-epithelialized with a residual scar measuring 2x2 cm | NR |
| **Full Thickness and Split Thickness Skin Graft** | | | | | | |
| Worley 2019^18^  Case report (N=2) | Differences in skin texture, contour, and color-match after FTSG on nasal tip (n=1) and below right eye (n=1) | AFL with CO2 (10,600 nm) and manual dermabrasion 2 months post-MMS | CO_2_RE Syneron, Candela  *Mode*: Fusion  *Ring*: 116,  *Fractional coverage*: 30%  *Core*: 70 mJ  *No. of passes:* 2 orthogonal passes  *No. of treatments:* 1 | N/A | Improvement of transition from the FTSG to the surrounding skin, scar texture, contour, and color-match to surrounding native skin | NR |
| Forbat 2017^19^  Case series (N=5) | Differences in skin texture, contour, and color-match after FTSG on the nose (n=1), nasal tip(n=1), nasal bridge (n=2), and nasal sidewall (n=1) | AFL with CO2 (10,600 nm) unspecified time post-MMS | UltraPulse, Lumenis  *Energy*: 100 -150 mJ  *Computer-pattern generator setting*: 1-2-5.  *No. of treatments:*1-2, 3-months apart | Endpoint was determined via achievement of flattening of the raised or uneven skin surface. | Improvement of transition from the FTSG to the surrounding skin, with improvement in contouring and an improvement in color in certain cases | NR |
| Verhaeghe 2013^20^  Prospective randomized,  evaluator-blinded, comparative split-scar study (N=24) | Scarring after FTSG (n=17), STSG (n=1), and primary closure (n=6); nose (n=15), then forehead (n=4), cheeks (n=3), perioral (n=1), neck (n=1) | NAFL (1 540nm), up to 4-years post-MMS | Starlux 300 with Lux fractional handpiece, Palomar Technologies  *Fluence:* 4.5-8.5 J/cm^2^, with increases of 0.5-1.0 J/cm^2^ at every session, depending on side effects  *Pulse duration:* 15 ms  *No. of passes:* 3-4  *No. of treatments:* 4, at 1-month intervals | PhGA via a visual analogue scale, PGA via a visual analogue scale, POSAS, and Skin reflectance measurements | PhGA score comparing the treated to the untreated control side was significantly different 1 (P=0.009) and 3 (P=0.001) months after treatment; statistically significant difference of the PGA was observed 1 (P < 0.001) and 3 (P < 0.001) months after treatment; significant difference of the total POSAS evaluation for the observer and patient part of the scale 1 and 3 months after treatment.  Skin reflectance measurements did not show significant difference | Erythema (67%), edema (31%), crusts (22%), burning sensation (14%), purpura (9%) and vesicles (4%) were reported 4-days after treatments; no reported long-term adverse |
| Ammirati 2001^21^  Case series (N=74) | Post-MMS defects on the tip (n=34), alar groove (n=21), sidewall (n=7), alar rim (n=7), and dorsum (n=5) ranging from 0.5 cm x 0.5 cm to 2.5 cm x 2.1 cm. | AFL with CO2 (10,600 nm) or long-pulsed Er:YAG lasers (2,940 nm), immediately post-MMS | CO2 with original SilkTouch attachment, Sharplan  *Energy:* 8–10 W  *Pulse duration:* 0.2-second  *Spot size:* 3–3.7 mm  CO2 with upgraded SilkTouch attachment, Sharplan  *Energy:* 10 W  *Spot size:* 3 mm  CO2 with FeatherTouch attachment, Sharplan  *Energy:* 40 W for contouring 10–14 W for *Energy:* feathering edges  *Spot size*: 3–5 mm  Cynosure, Chelmsford *Fluence:* 20–28 J/cm^2^  *Pulse duration:* 7–10 ms  *Spot size:* 3 mm | 3-point Cosmetic Evaluation Scoring Scale (n=30) | All 74 patients were satisfied with their results, and none have requested scar revision; of the 30 patients evaluated by the panel, all were scored acceptable to excellent | Mild hypo-pigmentation in at least 1 case |
| **Periscar Telangiectasia** | | | | | | |
| Villeneuve-Tang 2018^22^  Case series (N=6) | Periscar telangiectasia of the nose (n=5), and forehead (n=1) | KTP (532 nm) 3 months post-MMS | Excel V, Cutera  *Fluence:* 8.6–10.0 J/cm^2^  *Spot size*: 5-mm  *Pulse duration:* 15 ms  *No. of treatments:*1 | Severity of periscar telangiectasias assessed via a 3-point scale (mild, moderate, or severe); percent improvement of telangiectasias assessed via a 5-point scale (minimal to excellent); patient satisfaction assessed via a 5-point Likert-type scale | 4/6 of patients at least a 1-point improvement in the severity of telangiectasias; 2 patients had minimal clearance (0%–25%), 3 patients had good clearance (50%–75%), and 1 patient had excellent clearance (75%–100%); 3/6 patients reported at least a 1-point improvement in satisfaction | None reported. |
| **Functional Scar Contracture** | | | | | | |
| Ezra 2016^23^  Case series (N=10) | Hypertrophic scar (n=5); hypertrophic scar with ectropion (n=1); hypertrophic scar with eclabium (n=1); atrophic scar with ectropion (n=2); sclerotic scar (n=1) | Microsecond Nd:YAG laser (1,064 nm) 5-8 weeks post-MMS | Laser Genesis, Cutera  *Fluence:* 13 J/cm^2^  *Pulse duration*: 0.3 ms  *Repetition rate:* 4-10 Hz (300–1650 total pulses per treatment)  *No. of treatments:* 1-3, 1-2 months apart | Endpoint was determined via patient satisfaction with the appearance of their scars and/or resolution of any contractures that formed following surgery | All 10 patients pleased with the improved appearance of their scars; 4 patients had complete resolution of an ectropion or eclabium that formed secondary to scar contractures | 1–2 hours of erythema; no contractures |
| Ezra 2014^24^  Case series (N=4) | Lower lid ectropion (n=3), upper lip eclabium (n=1) | Microsecond Nd:YAG laser (1,064 nm) 6-8 weeks post-MMS | Laser Genesis, Cutera  *Fluence:* 13-14 J/cm^2^  *Pulse duration:* 0.3 ms  *Spot size*: 5mm  *Repetition rate*: 4 to 10 Hz  *No. of treatments:* 2 (for ectropion), 3 (for eclabium), at 2-month intervals.  *Irradiation pattern*: irradiation was applied in a zigzag fashion across the scar, 2 to 3 cm above the skin surface. | Endpoint was determined via no visible contracture in the treatment area, as determined by the dermatologic surgeon | Lower lid ectropion was resolved in 2/3 patients. Scar and ectropion improved in one patient after 2 treatment sessions; upper lip eclabium resolved in 1/1 patient | Erythema 1-2 hours post-treatment |
| **Scar Texture** | | | | | | |
| Chen 2020^26^  Case report (N=1) | Hypertrophic scar on the leg 3-months post-MMS | PDL (595 nm), 3 months post-MMS | Vbeam, Candela  *Fluence:* 14 J/cm^2^  *Spot size*: 7mm  *No. of treatments:*  2, 1-month apart | VSS and POSAS | 5-point improvement on VSS, 20-point improvement on patient SAS, 21-point improvement on observer SAS; 2 years later the scar had completely disappeared | NR |
| Kunishige 2010^25^  Case series (N=6) | Atrophic, hypertrophic, hyper and hypopigmented, and erythematous scars (n=6). No sites were specified. | NAFL (1,550 nm), unspecified time post-MMS | Fraxel, Reliant Technologies  *Energy:* 6 -70 mJ  *Density:* 312-2,500 microscopic treatment zones/cm^2^  *No. of passes:* 6-10 passes  *No. of treatments:* up to 8 treatments (average 3), at 1-month intervals until satisfaction with the clinical outcome was achieved. | Overall improvement, erythema, pigment and heights were graded using a quartile grading scale | Almost all patients achieved at least 50% improvement; hypertrophy, atrophy and erythema improved | Moderate to severe erythema and edema typically resolved within 24 to 48 hours. |
| Cohen 2013^27^  Case report (N=1) | Right cheek scar displaying telangiectasias, hypo- and hyper-pigmentation and isolated fibrotic lines | AFL with Er:YAG (2,940 nm), 2-3 months post-MMS | *Depth:* 150 – 200 microns  *Fractional Coverage*:  22%  N*o. of Passes:* 2  *No. of treatments:*  Three treatments spaced 4-5 weeks apart | Digital photography before initial laser session, 4-5 weeks after each of the 3 laser treatments and at 7 months postoperatively | Significant improvement in pigmentation, vascularity, and texture | NR |
| Schulz 2010^30^  Case report (N=1) | Atrophic scar on the right nasal ala due to secondary intention healing | NAFL (1,550 nm), 8-weeks post-MMS | Fraxel SR, Reliant Technologies  *Energy:* 40 mJ  *No. of passes:* 8 passes  *No. of treatments:* 5, at 1-month intervals | N/A | Scar nearly imperceptible 1-month post-treatment and remained stable 18-months post-treatment | Transient post-procedure erythema |
| Cohen 2016^31^  RCT (N=25) | Scars post-MMS on various parts of the body; face (n=22), chest (n=2), arm (n=1) | *Treatment arm 1:* PDL (595 nm), unspecified time post-MMS  *Treatment arm 2:* AFL with CO2 (10,600 nm), unspecified time post-MMS  Treatment arm 3: Combined PDL and AFL with CO2, unspecified time post-MMS  *Treatment arm 4:* one half of the scar was untreated (control), the other one half of the scar was treated with AFL with CO2 immediately after surgery and then 3 combined PDL and AFL with CO2 treatments | Vbeam Perfecta. Syneron, Candela (PDL)  *Fluence: 7.5* J/cm^2^  *Pulse duration*: 3 ms  *Spot size:* 10 mm  CO_2_RE Syneron, Candela (Fractional CO2 ablative laser)  *No. of treatments*: 3 (treatment arms 1, 2, and 3,) or 4 (treatment arm 4), 6-8 weeks apart | VSS and Global Evaluation Response Scale | Improvement in scar cosmesis was observed in all treatment arms according to the VSS and global evaluation response scale; in treatment arm 1, 2 patients had complete scar clearance and four patients had scars, which were almost cleared; in treatment arm 2, 4 patients’ scars almost cleared and two had moderate improvement; in treatment arm 3, 3 patients’ scars were almost cleared and one had moderate improvement; in treatment arm 4, 2 scars were almost cleared, compared to control, three had marked improvement and one had moderate improvement; improvement in vascularity was more noticeable following PDL than AFL with CO2, while AFL with CO2 demonstrated greater improvement in pigmentation | 74-91% of patients from all treatment arms reported none to moderate pain due to laser treatment. No adverse events were reported throughout the study |
| Kim 2014^32^  Prospective randomized,  evaluator-blinded, comparative split-scar study (N=14) | Scars on the face (n=12), and abdomen (n=2). | AFL with CO2 (10,600 nm) to one-half of participants’ scar and PDL (595 nm) to the other one-half of participants’ scar 2-weeks post-MMS | eCO2, Lutronic  *Energy:* 80 mJ *Spot density:* 100 spots/cm^2^  *Spot size:* 120 um  *Fractional Coverage:* 7.8%  *No. of Passes:* 2  *No. of treatments:* 3 over a 2-week period.  Vbeam, Candela  *Fluence:* 10 J/cm^2^  *Pulse duration:* 10 ms  *Spot size*: 7mm  *No. of treatments:* 3 over a 2-week period. | VSS | Both PDL and AFL with CO2 produced statistically significant improvements based on overall VSS (p<0.05), where overall 3.21-point improvement was observed with PDL and 2.50-point improvement  was observed with AFL with CO2.  However, there was no statistical difference between PDL and AFL with CO2  AFL with CO2 was more effective than PDL in the improvement of pliability and thickness. PDL was superior to AFL with CO2 in the improvement of vascularity and pigmentation | All reported post-therapy erythema and mild edema that resolved within 1 week. Post-treatment hyper-pigmentation developed in 2 patients who received with CO2 |
| Sobanko 2015^33^  Prospective,  evaluator- blinded, comparative, split scar- study (N=20) | >4cm linear scars on the forehead (n=5), temple (n=2), cheek (n=12), and nose (n=1) | AFL with CO2 (10,600 nm) to one-half of participants’ scar 6-7 days post-MMS | Ultrapulse Encore, Lumenis  *Energy:* 10 mJ  *Density:* 10%  *Spot size:* 7 mm  *No. of treatments:* 1 | VSS and patient cosmetic VAS | No statistically significant difference was observed in VSS (*P* = 0.31) by a blinded rater; a statistically significant difference observed in patient cosmetic VAS (*P* = 0.002), 12 weeks post-MMS | Transient redness of the treated site about 1 week after treatment (m =11) |
| Anolik 2011^34^  Retrospective chart review (N=10) | Facial non-melanoma skin cancers treated with MMS with healing by secondary intention (n=10). | Full-field erbium laser, immediately post-MMS | Not specified | Comparison of postoperative photographs 3–4 months after surgery as rated by blinded, non-treating dermatologists and evaluation of skin surface irregularities with the Primos optical tomography imaging system. | Patients undergoing laser ablation displayed less noticeable scars with less evident vertical ‘‘drop-off’’ and shadowing at the border of uninvolved to involved skin | Equal between treatment and non-treatment groups and included short-term side effects of erythema, swelling, and bruising |
| Tierney 2009^35^  Prospective randomized,  evaluator-blinded, comparative split-scar study (N=15) | Scars on the face (n=7), chest (n=5), neck (n=2), back (n=1). Keloids were excluded. | NAFL (1,550 nm) to one-half of participants’ scar and PDL (595 nm) to the other one-half of participants’ scar a minimum of 2-months post-MMS | Fraxel SR, Reliant Technologies  *Energy:* 70 mJ  *Fractional Coverage*:  23%  N*o. of Passes:* 16  *No. of treatments:* 4, at 2-week intervals  Vbeam, Candela  *Fluence*: 7.5 J/cm^2^  *Pulse duration*: 0.45 ms  *Spot size*: 10 x 3 mm | Scar dyspigmentation, thickness, texture, and overall cosmetic appearance were assessed on a 5-point grading scale | Significant improvements were noted in the portion of surgical scars treated with NAFL with respect to  pigmentation, thickness, texture, and overall cosmetic appearance  (Overall mean improvement 75.6%, range 60-100%, vs. PDL, 53.9%, range 20-80%; p<.001) | NAFL: mild to moderate pain during the treatment and mild post-treatment erythema and edema that resolved in 2 to 4 days; PDL: mild and limited to transient erythema and purpura |
| AFL: Ablative Fractional Laser  FTSG: Full-thickness skin graft  J: Joules  KTP: Potassium Titanyl Phosphate Laser  ms: millisecond  MMS: Mohs Micrographic Surgery  N/A: Not Available  NR: None Reported  NAFL: Non-Ablative Fractional Laser  PDL: Pulse Dye Laser  PGA: Patient's Global Assessment  PhGA: Physician Global Assessment  POSAS: Patient and Observer Scar Assessment Scale evaluation  RCT: Randomized Controlled Trial  STSG: Split Thickness Skin Graft  VAS: Visual Analog Scale | | | | | | |

**Identification of studies via databases and registers**

Records removed *before screening*:

Duplicate records removed (n = 382)

Records identified from (n=2529):

MEDLINE (n = 928)

EMBASE (n = 562)

Web of Science (n = 579)

Cochrane Library (n = 65)

CINAHL (n = 395)

**Identification**

Records screened

(n = 2147)

Records excluded

(n = 2115)

Reports sought for retrieval

(n = 34)

Reports not retrieved

(n = 0)

**Screening**

Reports assessed for eligibility

(n = 33)

Reports excluded (n=16):

Review article (n = 4)

Intraoperative lasers (n = 6)

Preoperative lasers (n = 2)

Conference abstract preceding published article (n = 4)

Studies included in review

(n = 17)

**Included**

**Figure 1:** Study selection. Preferred items for Systematic Reviews and Meta-Analyses (PRISMA) flow diagram. Out of 2147 identified studies and after application of the inclusion and exclusion criteria, 17 studies were included.
